# Human dengue virus serotype 2 neutralizing antibodies target two distinct quaternary epitopes

**DOI:** 10.1371/journal.ppat.1006934

**Published:** 2018-02-26

**Authors:** Emily N. Gallichotte, Thomas J. Baric, Boyd L. Yount, Douglas G. Widman, Anna Durbin, Steve Whitehead, Ralph S. Baric, Aravinda M. de Silva

**Affiliations:** 1 Department of Microbiology and Immunology, University of North Carolina at Chapel Hill School of Medicine, Chapel Hill, North Carolina, United States of America; 2 Department of Epidemiology, University of North Carolina at Chapel Hill School of Public Health, Chapel Hill, North Carolina, United States of America; 3 Johns Hopkins Bloomberg School of Public Health, Baltimore, Maryland, United States of America; 4 Laboratory of Infectious Diseases, National Institute of Allergy and Infectious Diseases, National Institutes of Health, Bethesda, Maryland, United States of America; University of Texas Medical Branch, UNITED STATES

## Abstract

Dengue virus (DENV) infection causes dengue fever, dengue hemorrhagic fever and dengue shock syndrome. It is estimated that a third of the world’s population is at risk for infection, with an estimated 390 million infections annually. Dengue virus serotype 2 (DENV2) causes severe epidemics, and the leading tetravalent dengue vaccine has lower efficacy against DENV2 compared to the other 3 serotypes. In natural DENV2 infections, strongly neutralizing type-specific antibodies provide protection against subsequent DENV2 infection. While the epitopes of some human DENV2 type-specific antibodies have been mapped, it is not known if these are representative of the polyclonal antibody response. Using structure-guided immunogen design and reverse genetics, we generated a panel of recombinant viruses containing amino acid alterations and epitope transplants between different serotypes. Using this panel of recombinant viruses in binding, competition, and neutralization assays, we have finely mapped the epitopes of three human DENV2 type-specific monoclonal antibodies, finding shared and distinct epitope regions. Additionally, we used these recombinant viruses and polyclonal sera to dissect the epitope-specific responses following primary DENV2 natural infection and monovalent vaccination. Our results demonstrate that antibodies raised following DENV2 infection or vaccination circulate as separate populations that neutralize by occupying domain III and domain I quaternary epitopes. The fraction of neutralizing antibodies directed to different epitopes differs between individuals. The identification of these epitopes could potentially be harnessed to evaluate epitope-specific antibody responses as correlates of protective immunity, potentially improving vaccine design.

## Author summary

Dengue viruses (DENV) are flaviviruses transmitted by mosquitos. There are approximately 390 million DENV infections every year, making dengue virus a major global public health concern. While there is a recently licensed DENV vaccine, it has low efficacy against preventing DENV2 infections. Individuals that are naturally infected with DENV2 generate neutralizing antibodies that can be protective against reinfection with DENV2. By studying three of these neutralizing antibodies, we found that they bind to two different locations on the surface of the virus. Additionally we found that most individuals that were naturally infected with DENV2, have antibodies circulating in their blood that target both of these regions. People who were vaccinated against DENV2 also make antibodies targeting both of these sites, suggesting they might also be protected against DENV2 infection. These studies reveal that human antibodies against DENV2 target the same two regions across multiple individuals. Additionally, for a DENV2 vaccine to be protective, it may be important to elicit antibodies directed to these regions as well.

## Introduction

Dengue virus (DENV) is a single stranded positive sense RNA virus that is transmitted by the *Aedes* mosquito [1]. There are four distinct DENV serotypes (DENV1-4), and infection results in a range of symptoms, from fever and rash to the more serious dengue hemorrhagic fever and dengue shock syndrome. Over a third of the world’s population is at risk for infection, and there are an estimated 390 million infections yearly [1]. A primary infection with DENV results in the induction of serotype cross-neutralizing antibodies which can provide temporary serotype cross-protective immunity that is not maintained [2]. Over the course of the following year, these cross-reactive neutralizing antibodies wane, leaving individuals susceptible to infection by the remaining three heterologous serotypes [3]. Serotype-specific neutralizing antibodies are maintained in circulation for decades following exposure and may play a critical role in providing subsequent protection against the infecting serotype [2,4,5]. While antibodies are known to play a key role in protection against DENV reinfection [6], it has also been shown that CD8+ T-cells [7,8], CD4+ T-cells [9], and other mechanisms of cellular immunity are important for protection [10,11].

The leading DENV vaccines are tetravalent formulations, designed to elicit independent, hopefully protective, neutralizing antibodies against all four serotypes simultaneously [12]. Phase 3 efficacy trials in Asia and Latin America showed that the recently licensed tetravalent vaccine, Dengvaxia, had variable efficacy depending on immune status prior to vaccination and the serotype of infection [13,14]. In mixed populations of susceptibles and DENV-immunes, Dengvaxia was 50–80% efficacious against DENV1, DENV3 and DENV4, but only 35–42% against DENV2 [13,14]. Vaccine efficacy was significantly lower in those persons seronegative to DENV compared to individuals who were DENV seropositive at the time of vaccination [13,14]. Moreover, younger vaccinated individuals were hospitalized for DENV more frequently than their unvaccinated counterparts, suggesting that poor immunogenicity in naïve subjects might place individuals at a greater risk of developing severe disease as antibody levels decline over time [15–18]. Indeed, based on long-term follow up data, Dengvaxia is no longer recommended for use in DENV-naive individuals [17]. The Dengvaxia clinical trials have revealed that even individuals with detectable neutralizing antibodies to a particular serotype experienced vaccine break-through infections indicating the mere presence of antibodies that neutralize infection in cell culture assays is not sufficient for protection [19]. Therefore, in addition to the level of neutralizing antibodies to each serotype, it is critical to define other properties of human antibodies potentially responsible for durable, protective immunity. Importantly, while Dengvaxia contains the structural proteins from DENV, the non-structural proteins are from yellow fever virus. It is therefore possible that sufficient T-cell immunity towards DENV epitopes was not achieved [16,20]. DENV vaccines that contain DENV non-structural proteins might generate a more robust T-cell response, and therefore more closely resemble a natural DENV infection, which results in a protective immune response [21].

**Fig 1:**
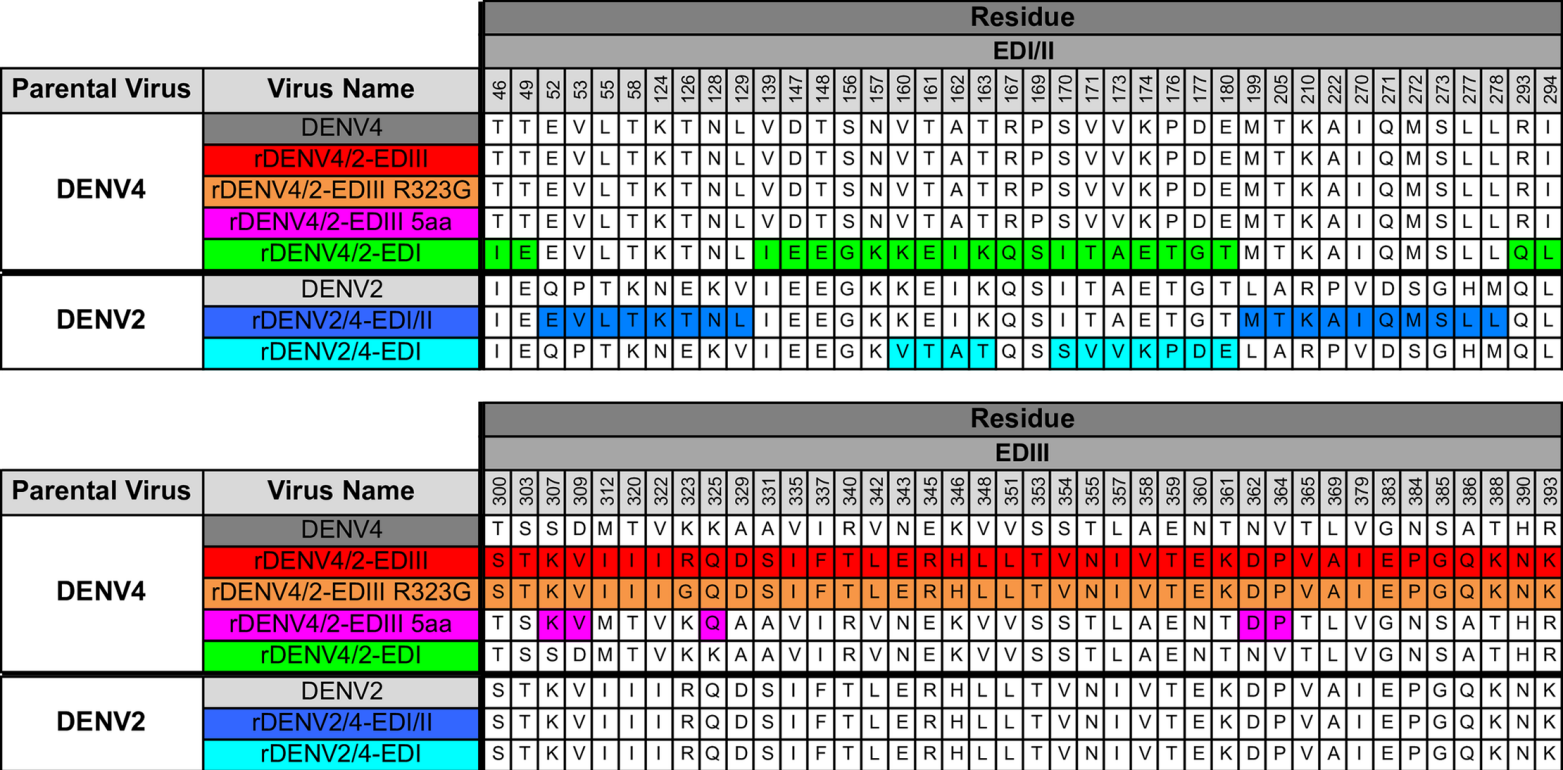
Sequences of rDENVs. Amino acid sequences of WT and rDENVs used to map antibody epitopes.

The DENV envelope glycoprotein (E) ectodomain, which is comprised of three domains (EDI, EDII and EDIII) is the major target of neutralizing antibodies [22]. Two E monomers form a dimer in a head-to-tail arrangement, three dimers form a raft, and thirty rafts (180 monomers) cover the entire surface of the virus [23]. Our group has previously characterized components of the antibody response to DENV1, DENV2, DENV3 and DENV4 infection by mapping the epitopes of strongly neutralizing human monoclonal antibodies (hMAbs) [24,25]. Importantly, many of these strongly neutralizing hMAbs target quaternary structure epitopes that form as the envelope glycoprotein is assembled on the virus surface [24–31]. In addition, we have demonstrated that we can transplant these quaternary epitopes between DENV serotypes and maintain their biological functions [25,30,32,33]. While determining the properties of individual hMAbs is valuable, complex polyclonal antibody response governs protection against subsequent infection. Importantly, the epitope of a single DENV2 serotype-specific hMAb, 2D22, was shown by our group to be targeted by a large fraction of DENV2 neutralizing antibodies in many, but not all individuals after recovery from DENV2 infections, highlighting the potential role of this epitope in protective immunity [25]. Despite this, there are additional DENV2 hMAbs that use other epitopes within E, suggesting that there are potentially multiple neutralizing antibody epitopes for each serotype. The goals of this study are to identify novel neutralizing epitopes in DENV2, to develop robust diagnostic reagents for evaluating epitope specific responses with recombinant DENVs (Fig 1) and to evaluate the role of these novel and previously described epitopes as targets of polyclonal serum antibodies induced by natural infections and DENV vaccines.

## Results

### Human monoclonal antibodies target quaternary epitopes on DENV2

**Fig 2:**
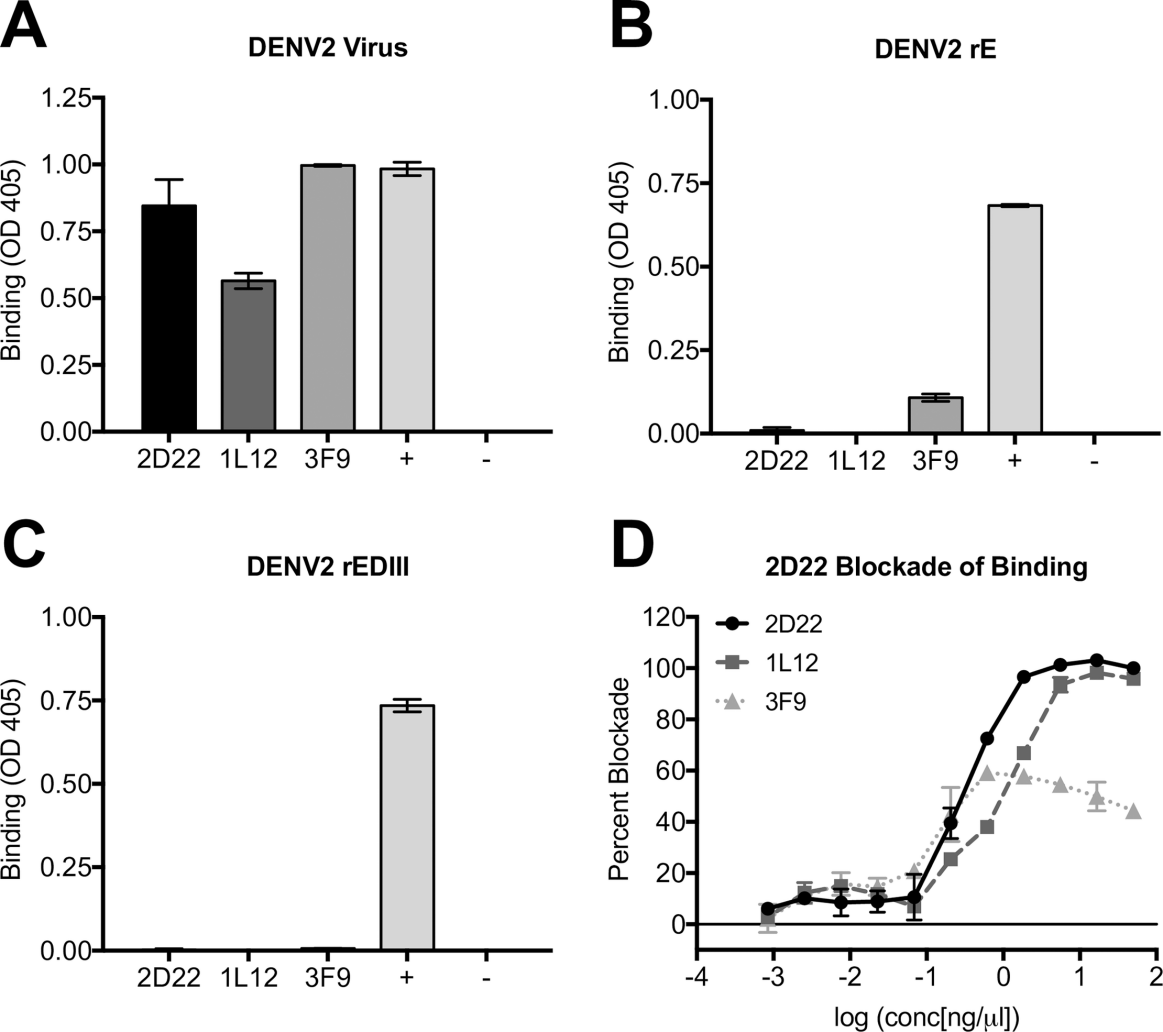
DENV2 serotype-specific hMAbs use multiple quaternary epitopes. DENV2 hMAbs 2D22, 1L12 and 3F9 were assessed for their ability to bind whole DENV2 virions (A), DENV2 rE (B), and DENV2 rEDIII (C). Positive control (+) hMAb is DVC10.16, a DENV2 hMAb that uses a simple A-strand epitope contained entirely within EDIII and 5J7 is a DENV3 serotype-specific hMAb as a negative control (-). (D) Blockade of binding assay where hMAbs 2D22, 1L12 or 3F9 were assessed for their ability to block 2D22-AP from binding to DENV2.

To characterize the epitopes of DENV2 human monoclonal antibodies (hMAbs), we used a panel of three DENV2-specific, strongly neutralizing hMAbs (S1 Table). The hMAbs were isolated from two donors infected in geographically distinct locations with different DENV2 genotypes [34]. The three hMAbs, 3F9, 2D22 and 1L12, bound to whole DENV2 virus (Fig 2A). Recently, it has been reported that human antibodies that strongly neutralize DENVs bind to quaternary structure epitopes displayed on E homo-dimers or higher order surface structures required for virion assembly [24,25,29,35]. Consonant with previously published results, DENV2 hMAb 2D22 did not bind rE or rEDIII, confirming the quaternary epitope specificity (Fig 2B and 2C). HMAb 1L12 was similar and did not bind to rE or rEDIII (Fig 2B and 2C). In contrast, hMAb 3F9 weakly bound to rE (Fig 2B). Because hMAb 3F9 bound well to DENV2 virions and weakly to rE, it is likely that the epitope is dependent on E protein assembly into virions for optimal display. In a blockade of binding assay, 2D22 interfered with 1L12 for binding to DENV2, suggesting that they recognize proximal or overlapping epitopes on the viral envelope (Fig 2D). In contrast, 3F9 only partially blocked the binding of 2D22 (Fig 2D) indicating the two hMAbs recognize distinct epitopes on the viral envelope.

### HMAbs 2D22 and 1L12 bind to proximal but distinct epitopes

**Fig 3:**
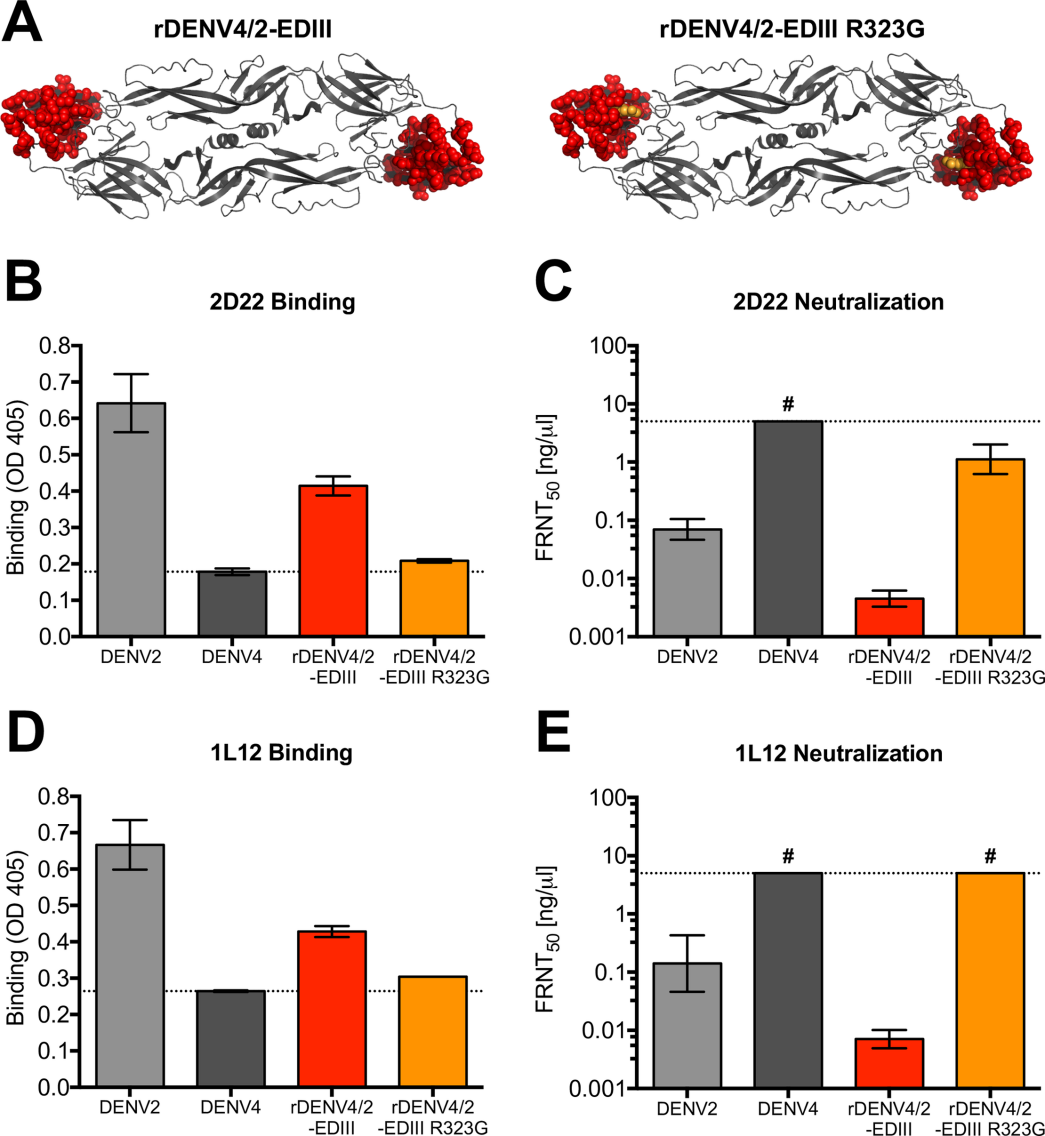
HMAbs 2D22 and 1L12 use EDIII in their epitopes. (A) rDENV4/2-EDIII is DENV4 virus containing entire EDIII from DENV2. rDENV4/2-EDIII R323G is rDENV4/2-EDIII virus with single point mutation at residue 323. 2D22 and 1L12 were assessed for their ability to bind (B and D) and neutralize (C and E) recombinant DENVs in ELISA binding assays and Vero-81 Focus Reduction Neutralization Tests (FRNT). Dotted line in ELISA represents the background signal. FRNT_50_ represents the concentration of antibody required to neutralize 50% of infection. # = virus was not neutralized at highest concentration of hMAb tested (5ng/μl).

The cryo-EM structure of hMAb 2D22 Fab in complex with DENV2 has been solved [28] and the footprint of the antibody spans EDIII and EDII of two E molecules forming each homo-dimer. Although 2D22 did not bind rEDIII (Fig 2C), the antibody binds and neutralizes a DENV4 virus containing the entire EDIII from DENV2 (rDENV4/2-EDIII) (Fig 3) [25,28]. Introducing a single point mutation into this virus (rDENV4/2-EDIII R323G), previously identified as a 2D22 escape mutation [24], (Fig 3A), resulted in a loss of binding and neutralization (Fig 3B and 3C), confirming 2D22 uses the transplanted EDIII region. HMAb 1L12, which was isolated from a different donor, showed nearly identical phenotypes, where it gained binding and neutralization to rDENV4/2-EDIII, indicating that it uses EDIII as part of its complex quaternary epitope (Fig 3D and 3E). Similarly, the R323G mutation in rDENV4/2-EDIII results in complete loss of 1L12 binding and neutralization (Fig 3D and 3E).

**Fig 4:**
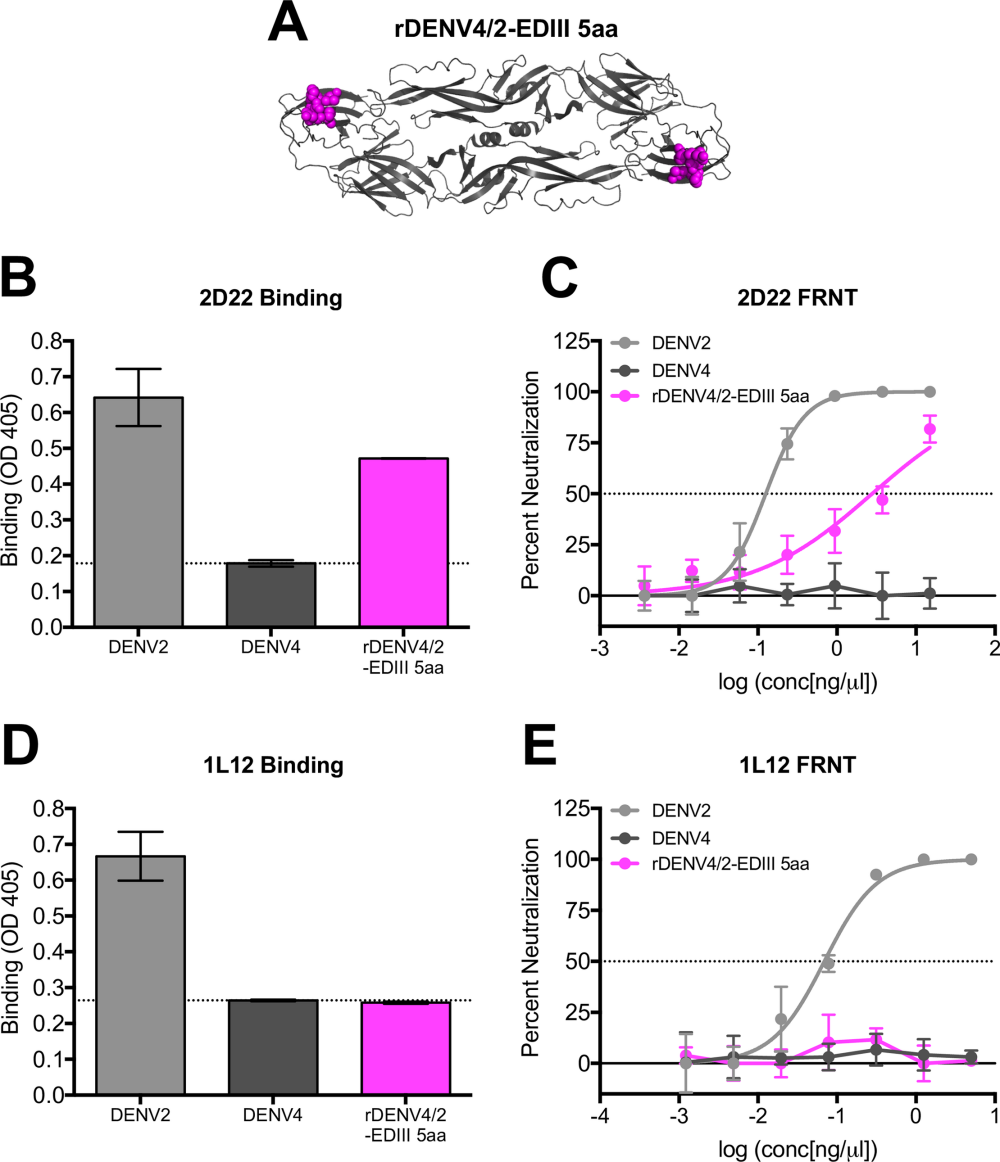
HMAbs 2D22 and 1L12 use different critical residues in their epitopes. (A) rDENV4/2-EDIII 5aa is a DENV4 virus with five EDIII residues from DENV2. 2D22 and 1L12 were assessed for their ability to bind (B and D) and neutralize (C and E) recombinant DENV in ELISA binding assays and Vero-81 Focus Reduction Neutralization Tests (FRNT). Dotted line in ELISA represents the background signal, determined as the OD value of wells containing all reagents except for viral antigen.

In addition to binding highly conserved residues in EDII, cryo-EM studies predict that hMAb 2D22 interacts with eight (307, 309, 310, 316, 318, 362, 363, 364) surface-exposed amino acids in DENV2 EDIII [28], five (307, 309, 316, 362, 364) of which differ between DENV2 and DENV4. To refine the map coordinates of 2D22 and 1L12 epitopes, we generated a new EDIII recombinant virus in which these five amino acids in DENV4 were replaced with those from DENV2 (rDENV4/2-EDIII 5aa) (Fig 4A). 2D22 was able to partially bind and neutralize this virus at high concentrations of antibody (Fig 4B and 4C). Because the gain in function is only partial, these data suggest that the epitope requires other critical residues in EDIII for maximal binding and neutralization. In contrast, hMAb 1L12 did not bind or neutralize rDENV4 –EDIII 5aa (Fig 4D and 4E), suggesting that its epitope overlaps with 2D22 but engages a different set of residues on EDIII.

### 3F9 targets a complex EDI epitope

**Fig 5:**
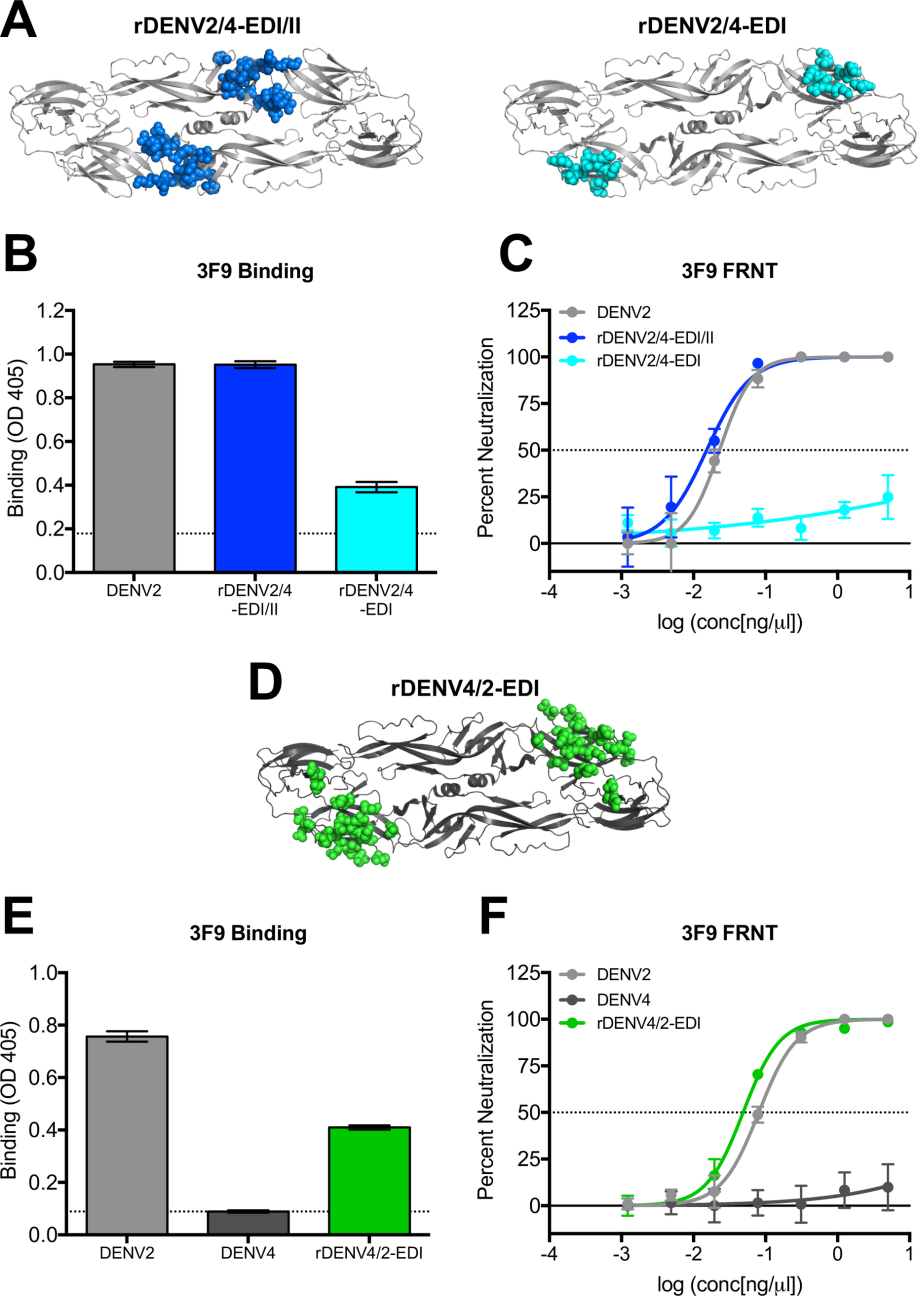
HMAb 3F9 use an epitope contained within EDI. (A) rDENV2/4-EDI/II is DENV2 virus containing EDI/II hinge region residues from DENV4. rDENV2/4-EDI is DENV2 virus containing EDI residues from DENV4. 3F9 was assessed for its ability to bind (B and E) and neutralize (C and F) recombinant DENVs in ELISA binding assays and Focus Reduction Neutralization Tests (FRNT) in Vero-81 cells (C) or C6/36 cells (F). Dotted line in ELISA represents the background signal. (D) rDENV4/2-EDI is DENV4 virus containing EDI residues from DENV2.

Competition assays with 2D22 indicated that 3F9 binds to an epitope that has minimal if any overlap with 2D22 or IL12 (Fig 2D). To map the epitope of hMAb 3F9, we evaluated its binding to a panel of chimeric recombinant DENVs (rDENVs) with alterations in specific domains (Fig 5A). Our group has previously shown that strongly-neutralizing hMAbs for DENV1 and DENV3 use the EDI/II hinge region in their epitope [24,26,27,32]. hMAb 3F9 bound to a DENV2 virus that had the EDI/II hinge residues replaced with those from DENV4 (rDENV2/4-EDI/II), suggesting it does not use this region in its epitope (Fig 5B). Conversely, hMAb 3F9 lost most binding to and neutralization of a DENV2 with 11 of its EDI residues replaced with those from DENV4 (rDENV2/4-EDI), suggesting 3F9 uses an epitope that contains the replaced residues located in EDI (Fig 5B and 5C). To further characterize the 3F9 epitope, we tested its binding to and neutralization of a DENV4 virus that contained 22 surface-exposed EDI residues from DENV2 (rDENV4/2-EDI) (Fig 5D). 3F9 bound to and neutralized the EDI transplant virus, confirming EDI as the main target of this hMAb (Fig 5E and 5F), however gain of binding was not complete, suggesting there are other residues that are required for maximal binding. Our data underscore the importance of cryo-EM analyses to help elucidate the complete 3F9 binding epitope.

In summary, these studies define the location of epitopes recognized by DENV2 type-specific neutralizing hMAbs 2D22, 1L19 and 3F9. Both 2D22 and 1L19 bind to proximal but distinct quaternary epitopes centered on EDIII. hMAb 3F9, on the other hand, binds to an epitope on EDI of the E protein.

### DENV2 polyclonal neutralizing antibodies target epitopes defined by hMAbs

**Fig 6:**
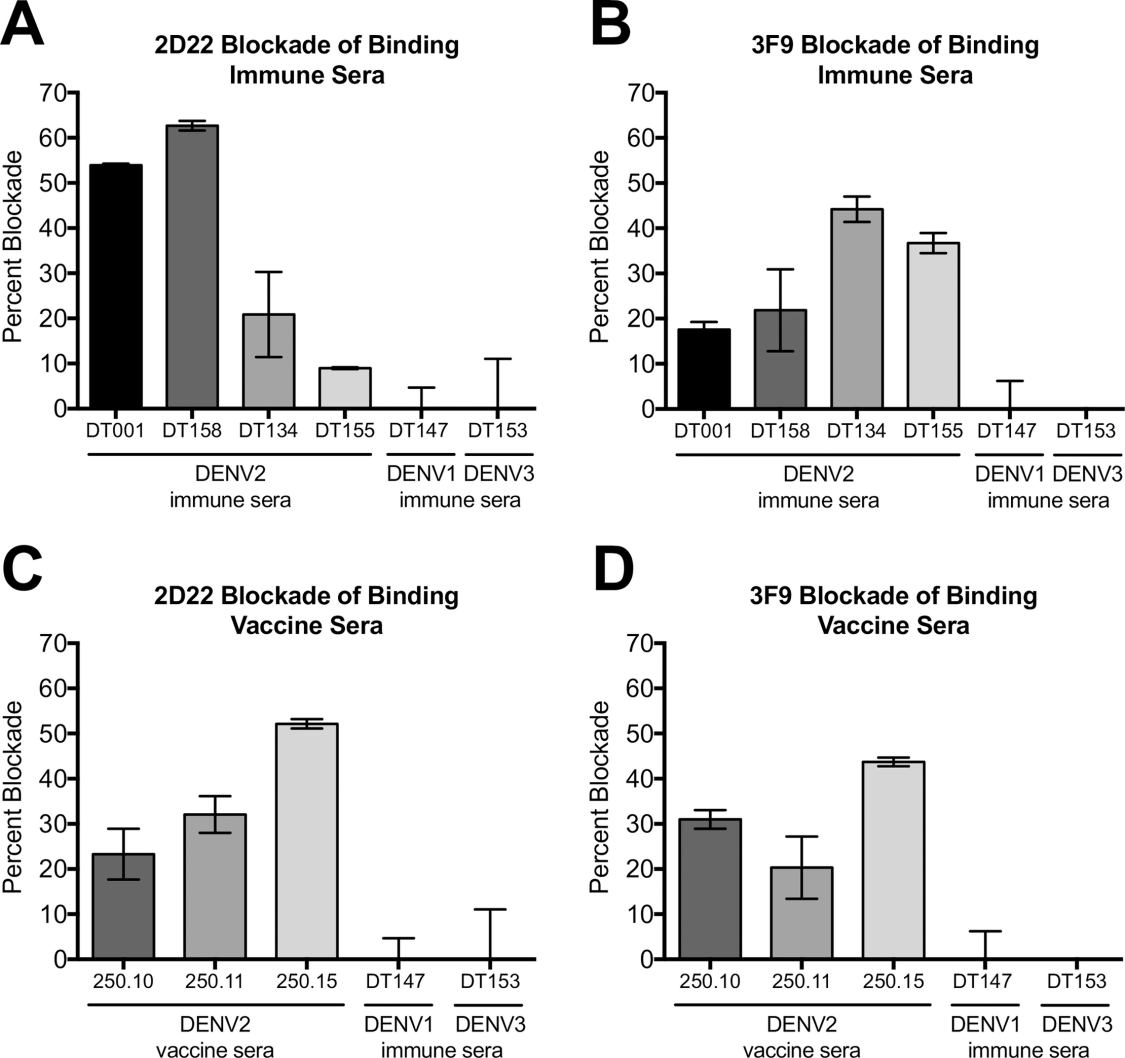
DENV2 polyclonal antibodies target EDIII and EDI epitopes. Blockade of binding assay where DENV2 natural infection immune sera (A, B) or DENV2 monovalent vaccine sera (C, D), were assessed for their ability to block 2D22-AP (A, C) or 3F9-AP (B, D) from binding to their respective epitopes on DENV2. DENV immune sera were depleted of cross-reactive antibodies prior to blockade assay.

To determine if epitopes defined using hMAbs were targets of polyclonal serum neutralizing antibodies, we first performed competition (blockade of binding) assays with human immune sera and hMAbs. Convalescent immune sera from primary DENV2 cases effectively blocked the binding of 2D22 to its epitope (Fig 6A). Under identical conditions of treatment, DENV1 or DENV3 immune sera did not block 2D22 from binding, confirming that primary DENV2 infection elicited a 2D22-like serotype-specific antibody response (Fig 6A). The same DENV2 immune sera also blocked 3F9 from binding to its epitope, whereas control DENV1 and DENV3 sera did not (Fig 6B). Remarkably, the ratio of antibodies targeting the two epitopes appeared to differ across individuals. Two individuals (DT001 and DT158) were more effective at blocking 2D22 binding than 3F9 binding, whereas DT134 and DT155 were more effective at blocking 3F9 than 2D22 (Fig 6A and 6B), suggesting these individuals had different ratios of antibodies targeting each epitope. DENV2 monovalent vaccine sera also blocked 2D22 and 3F9 binding to their respective epitopes (Fig 6C and 6D), indicating that natural infection and monovalent DENV2 vaccine-elicited antibodies target both these epitopes. Interestingly, no DENV2 sera samples were able to completely inhibit either 2D22 or 3F9 from binding to their epitopes, suggesting a limit in the amount these blocking antibodies are present in the sera.

**Fig 7:**
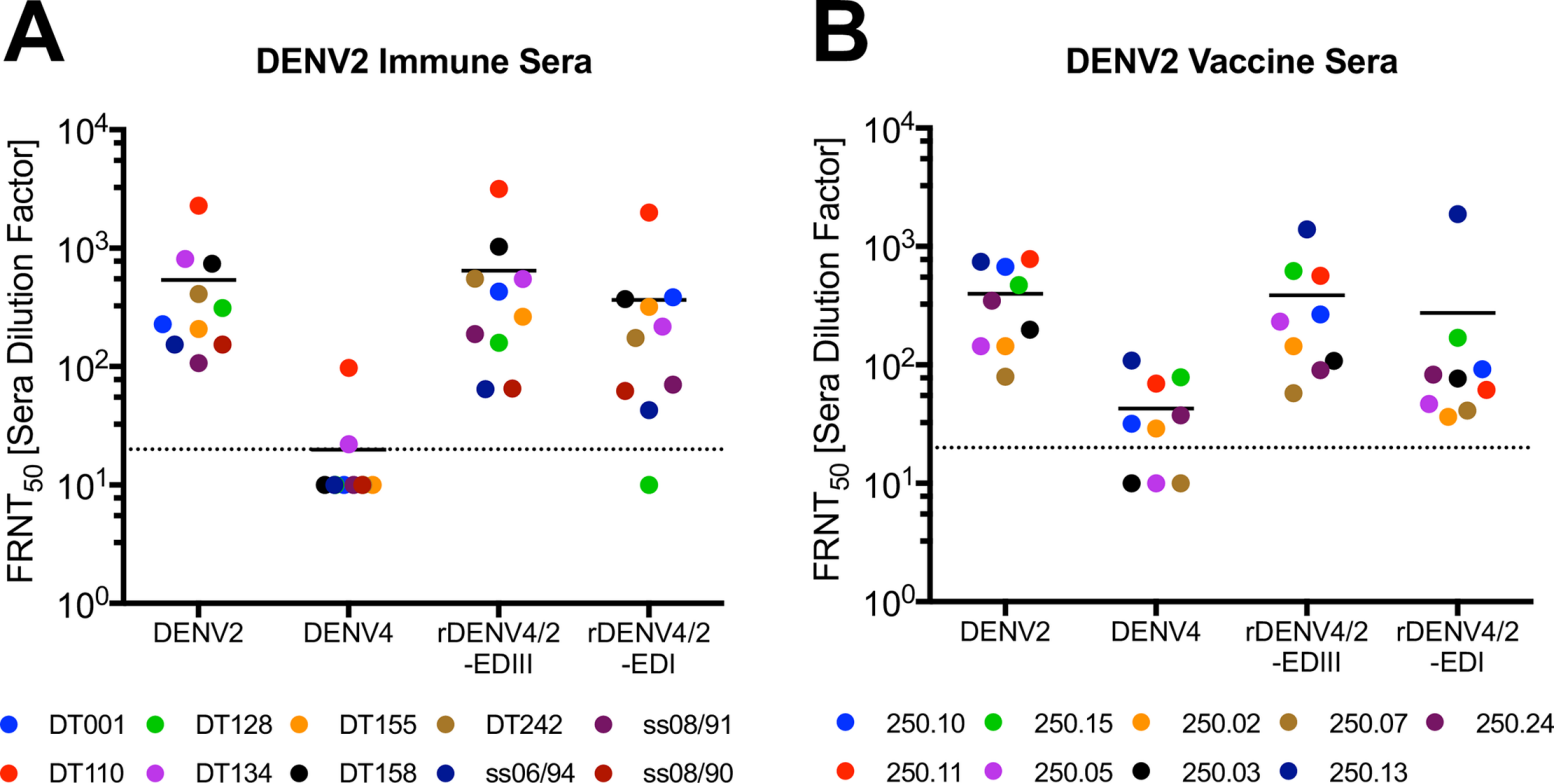
DENV2 polyclonal neutralizing antibodies target two distinct epitopes. DENV2 immune sera (A) and monovalent vaccine sera (B) were evaluated for their ability to neutralize WT and rDENV in C6/36 Focus Reduction Neutralization Tests (FRNT). Dotted line represents limit of detection (20), samples with no neutralization were plotted at one half the limit of detection (10). Y-axis indicates sera dilution factor required to neutralize 50% of virus.

Next, we performed studies to determine if 2D22 and 3F9 epitopes were targets of DENV2 neutralizing serum antibodies. Epitope exchanged recombinant viruses not only provide an approach to map hMAbs, but they can also be used to quantify epitope-specific neutralizing antibodies in immune sera. To measure the amount of neutralizing antibodies targeting 2D22 and 3F9 epitopes, we evaluated the ability of polyclonal DENV2 immune sera (10 samples) or vaccine sera (9 samples) to neutralize rDENV4/2-EDIII (Fig 3A) and rDENV4/2-EDI (Fig 5D) viruses. Consistent with previous results [25], a large fraction of DENV2 neutralizing antibodies tracked with DENV2 EDIII displayed on the rDENV4/2-EDIII virus (Fig 7A). Interestingly, most individuals also had neutralizing antibodies that tracked with the DENV2 EDI epitope displayed on the rDENV4/2-EDI virus (Fig 7A). In some individuals (e.g. DT155) there are similar levels of neutralizing antibodies that target both epitopes, whereas in other individuals (e.g. DT128) few if any neutralizing antibodies target the 3F9 EDI epitope (Fig 7A). In individuals that received a monovalent DENV2 vaccine, the majority of their neutralizing antibodies target EDIII with a much smaller fraction of the response targeting the EDI epitope (Fig 7B). Overall, there is higher tracking of DENV2 specific responses with both the EDIII and EDI epitopes (80% and 54% respectively) in the natural infection sera, as compared with the vaccine sera (69% and 30% respectively), suggesting vaccination elicits a slightly different antibody response (Table 1).

**Table 1:** Percent polyclonal neutralization tracking with each epitope.

		FRNT_50_ [Sera Dilution Factor] ^α^					Percent tracking with ^β^	
Type	Sera ID	DENV2	DENV4	rDENV4/2-EDIII	rDENV4/2-EDI		EDIII	EDI
**Natural Infection Sera**	DT001	228	---	431	385		100	100
	DT110	2286	97	3170	2004		100	88
	DT128	313	---	158	---		51	0
	DT134	809	22	550	217		65	24
	DT155	208	---	263	320		100	100
	DT158	740	---	1030	372		100	50
	DT242	410	---	553	175		100	43
	ss06/94	153	---	65	43		42	28
	ss08/91	107	---	188	70		100	66
	ss08/90	154	---	65	62		42	41
	*Average ±SD*	* *	* *	* *	* *	* *	*80% ± 27*	*54% ± 34*
								
**DENV2 Monovalent Vaccine Sera**	250.10	675	32	267	92		35	9
	250.11	783	69	566	62		63	0
	250.15	473	79	621	169		100	19
	250.05	144	---	232	47		100	33
	250.02	144	29	144	36		80	5
	250.03	199	---	108	77		55	39
	250.07	80	---	58	41		72	52
	250.13	743	109	1396	1871		100	100
	250.24	348	38	90	83		15	13
	*Average ±SD*						*69% ± 30*	*30% ± 31*

^α^ FRNT_50_ [Sera Dilution Factor] were calculated as the sera dilution factor required to neutralize 50% of the virus, extrapolated from Fig 7.

^β^ Percentage calculated as = (rDENV4/2-EDIII FRNT_50_ –DENV4 FRNT_50_)/(DENV2 FRNT_50_) x 100, and = (rDENV4/2-EDI FRNT_50_ –DENV4 FRNT_50_)/(DENV2 FRNT_50_) x 100.

## Discussion

People infected with DENVs develop robust and durable antibody responses that contribute to protection against re-infection against the homologous serotype; however, rare instances of re-infection with the same serotype do occur [36]. Antibodies that neutralize DENVs in cell-culture assays have been considered to be surrogates of protective immunity *in vivo*. However, this assumption has been challenged by recent results from DENV vaccine trials. Most notably, people who received a tetravalent live attenuated DENV vaccine and developed neutralizing antibodies experienced DENV2 breakthrough infections [15]. Breakthrough infections were also documented with the other serotypes despite the presence of neutralizing antibodies [15]. This landmark vaccine trial has established that the presence of cell-culture neutralizing antibodies identified using FRNT assays, is not predictive of protection. Indeed, breakthrough DENV infections of vaccinated seronegative children underscore the urgency to understand the essential mechanisms of immune protection in DENV. Moving forward, we need to define key epitopes on DENVs targeted by neutralizing and potentially protective antibodies and develop assays to measure both the level and the molecular specificity of neutralizing antibodies.

In this study, we used a panel of hMAbs, human DENV polyclonal immune sera, and recombinant DENVs (Fig 1) to map the location of epitopes recognized by DENV2 neutralizing antibodies. First, we used three DENV2 type-specific and strongly neutralizing hMAbs to map epitopes. hMAbs 2D22 and 1L12 isolated from different people had similar properties and recognized overlapping quaternary epitopes centered on EDIII. Recently Fibriansah *et*. *al*. determined the cryo-EM structure of 2D22 bound to DENV2 and demonstrated that the footprint of the 2D22 spanned EDIII and EDII of two E proteins forming a single homo-dimer [28]. Our data indicating that 2D22 recognizes an EDIII centered quaternary epitope are entirely consistent with the footprint determined by Fibriansah *et*. *al*. We suspect that 1L12 also binds a similar but not identical epitope because of subtle differences in the binding of 2D22 and 1L12 noted in this study. These findings highlight the importance of cryo-EM studies with IL12, which would provide a more comprehensive view of this larger DENV2 antigenic site. Nevertheless, our observation that two individuals infected with different DENV2 genotypes produced type-specific neutralizing hMAbs targeting a similar region suggests that EDIII is a dominant target of DENV2 neutralizing antibodies. The DENV1, 3 and 4 type-specific, neutralizing hMAbs identified to date do not map to the regions defined by 2D22 and 1L12 indicating that major targets to type-specific neutralizing Abs can differ between serotypes. However, several DENV serotype cross-neutralizing hMAbs that bind across the E homo-dimer have been described recently [29]. While these E dimer-dependent epitope (EDE) hMAbs partially overlap with the 2D22 epitope, they recognize patches that are highly conserved between serotypes unlike 2D22.

HMAb 3F9 and 1L12, which were isolated from the same person, have distinct epitopes, consistent with bivalent recognition of the EDIII and EDI DENV epitopes in most DENV polyclonal immune sera. The 3F9 epitopes is centered on EDI at a site that overlaps with known DENV1 and DENV4 neutralizing hMAbs [26,37,38]. Therefore, unlike 2D22, the region recognized by 3F9 is targeted by type-specific neutralizing antibodies to other serotypes as well.

Our previous work demonstrated that a majority of the polyclonal antibody response following DENV2 infection and vaccination appeared to be directed to a quaternary EDIII epitope [25]. In some individuals however, neutralization titers did not track as strongly with this epitope, suggesting that two or more neutralizing epitopes are targeted disproportionately after primary DENV2 infections. We propose that the EDI epitope defined by the hMAb 3F9 represents a second major neutralizing epitope on DENV2. Most individuals with naturally acquired DENV2 infections contained antibodies targeting both epitopes however some individuals targeted only one epitope, or had a skewed response. Similar results were observed in DENV2 vaccinated individuals, where there were antibodies targeting each epitope, however the overall response is dominant to the EDIII epitope. Overall, there was a higher response of antibodies tracking with the EDIII than the EDI epitope in both the natural infection and vaccinated sera (Table 1). Interestingly, some individuals had complete neutralizing antibody responses tracking with both epitopes, suggesting that they potentially generated redundant populations of antibodies. Generating populations of antibodies directed to different regions on E could be an important component of an effective antibody response. Viruses can mutate to escape antibody pressure, but simultaneously escaping antibody pressure to multiple sites on E would be more challenging [39,40]. As some individuals appear to mount preferential responses to one site or the other after natural infection or vaccination, it is possible that strains with natural variation within one of these epitopes may allow for repeat or breakthrough DENV2 infections.

Without a clear understanding of what constitutes a protective DENV antibody response to each serotype, it is challenging to evaluate current DENV vaccines. By defining the epitopes targeted by DENV2 hMAbs and polyclonal sera, we hope to determine if there are antibody based correlates of protection and use these to evaluate current vaccines in the pipeline, and inform the design of next-generation vaccines. Using recombinant DENVs that contain both gain of function and loss of function epitopes, we can rapidly map in high-throughput assays the epitopes of large panels of hMAbs, prioritizing targets for crystallographic studies and downstream analyses.

## Methods

### Virus construction

Recombinant viruses were constructed using a four-cDNA cloning strategy. The DENV genome was divided into four fragments, and subcloned into separate cDNA plasmids with unique type IIS restriction endonuclease cleavage sites at the 5’ and 3’ ends of each fragment. A T7 promoter was introduced into the 5’ end of the A fragment. Plasmid DNA was grown in *Escherichia coli* cells, digested with the corresponding enzymes, gel purified, ligated together with T4 DNA ligase and transcribed with T7 polymerase to generate infectious genome-length capped viral RNA transcripts. RNA was electroporated into C6/36 cells, cell culture supernatant containing virus was harvested and passaged onto C6/36 cells to generate a passage one virus stock.

### Cells

C6/36 cells (ATCC CRL-1660) were grown in Gibco minimal essential medium (MEM) at 32°C. Vero-81 cells (ATCC CCL-81) were maintained in Dulbecco’s modified Eagle’s medium (DMEM) at 37°C. Media were supplemented with fetal bovine serum (FBS) (10% for Vero-81 and 5% for C6/36) which was lowered to 2% after infection. C6/36 media were supplemented with nonessential amino acids. All media were additionally supplemented with 100U/ml penicillin, 100μg/ml streptomycin and 0.25μg/ml Amphotericin B. All cells were incubated in 5% CO_2_.

### Ethics statement

Human dengue immune sera used in this study were obtained from a previously described Dengue Traveler collection at University of North Carolina, and were all primary DENV2 natural infections [24,25,30]. Vaccine sera were obtained from individuals who received a live-attenuated monovalent DENV2 vaccine as developed by the US National Institutes of Health (NIH) and were provided by Anna Durbin and Stephen Whitehead. All human sera samples were obtained under Institutional Review Board approval and were anonymized.

### Virus titration and immunostaining

One day prior to inoculation, 24-well cell culture plates were seeded with 5x10^4^ Vero-81 cells. Virus stocks were serially diluted 10-fold then added to cells (after growth media was removed) for one hour at 37°C. After incubation, cells were overlaid with 1% methylcellulose in OptiMEM I (Gibco) supplemented with 2% FBS, nonessential amino acids and 100U/ml penicillin, 100μg/ml streptomycin and 0.25μg/ml Amphotericin B, and incubated at 32°C. After four days incubation, overlay was removed, cells were washed with phosphate-buffered saline (PBS) and fixed in 80% methanol. Cells were blocked in 5% non-fat dried milk (blocking buffer) then incubated with anti-prM MAb 2H2 and anti-E MAb 4G2 diluted in blocking buffer. Cells were washed with PBS, then incubated with horseradish peroxidase (HRP)-conjugated goat anti-mouse antibody (Sigma) diluted in blocking buffer. Plates were washed and foci were developed using TrueBlue HRP substrate (KPL).

### Binding Enyzme-Linked Immunosorbent Assay (ELISA)

For whole DENV ELISA, plates were coated with 100ng/well mouse MAb 4G2 and 2H2 overnight at 4°C. Plates were washed with Tris-buffered saline with 0.05% Tween (TBST) and blocked in 3% non-fat dried milk in TBST (blocking buffer), and equal quantities of virus (as previously titrated by ELISA using cross-reactive polyclonal DENV immune sera) were added and incubated for 1 hour. For rE and rEDIII ELISA, plates were directly coated with protein and incubated. Plates were washed and primary human MAbs were diluted in blocking buffer and added to plate for 1 hour. Plates were washed and alkaline phosphate (AP)-conjugated secondary antibodies were added for 1 hour. Plates were washed, developed using p-nitrophenyl phosphate substrate and color changes were quantified by spectrophotometry. Assays were developed until OD values were within linear range of the assay, therefore absolute OD values may vary between graphs. All binding assays are based on two experiments performed in duplicate.

### Blockade of binding assay

Plates were coated with antibody, blocked, and virus was captured as described above. DENV polyclonal immune sera were depleted of cross-reactive antibodies as described previously [30]. Briefly, sera were incubated with beads coated with purified DENV4 antigen, then beads were pelleted to removed cross-reactive antibodies bound to bead:antigen complexes. Cross-reactive depleted sera were then diluted 1:10 in blocking buffer and incubated for 1 hour. Plates were washed and alkaline phosphate (AP)-conjugated 2D22 (100ng/well) or 3F9 (50ng/well) were added for 1 hour. Plates were developed as described above. Percent blockade was calculated as follows = (100-[OD of sample/OD of negative control]*100). Blockade of binding assays are based on two experiments performed in duplicate.

### Focus reduction neutralization test

For the focus reduction neutralization test (FRNT), hMAbs were diluted 4-fold and mixed with ~45 focus-forming units (FFU) of virus, and incubated for 1 hour at 37°C. After incubation, virus:hMAb mixture was added to Vero-81 cells for 1 hour at 37°C or C6/36 cells for 1 hour at 32°C, then overlay was added and cells were incubated and fixed and stained as described above. Foci were counted and FRNT_50_ titers were calculated as the concentration of antibody or sera dilution factor required to neutralize 50% of the virus. Neutralization assays are based on two (HMAbs) or one (immune sera) experiments performed in triplicate.

## Supporting information

S1 Table DENV2 monoclonal antibodies. Virus was isolated from subject DT001, sequenced and found to be part of the cosmopolitan genotype [41]. Subject IRB019 was infected in Thailand in 1997 when the DENV2 Asian genotype strain was circulating in the region.
(TIF)
