# Supplementary figures and images for: Human dengue virus serotype 2 neutralizing antibodies target two distinct quaternary epitopes
Source: PLoS Pathog. 2018 Feb 26;14(2):e1006934. doi: 10.1371/journal.ppat.1006934 (PMC5843351; doi:10.1371/journal.ppat.1006934)

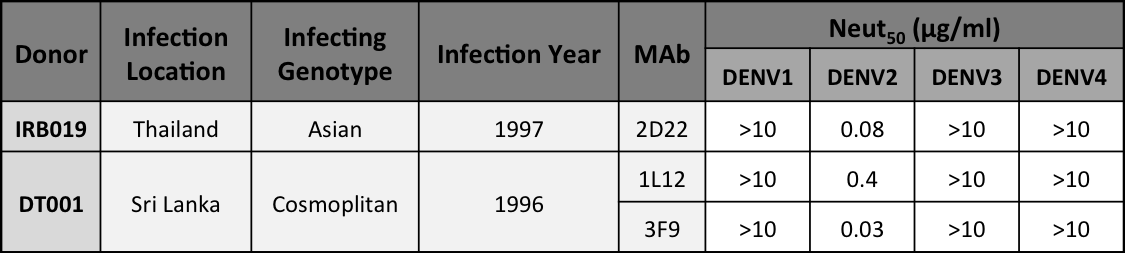

Supplement: S1 Table — Virus was isolated from subject DT001, sequenced and found to be part of the cosmopolitan genotype [41]. Subject IRB019 was infected in Thailand in 1997 when the DENV2 Asian genotype strain was circulating in the region. (TIF) [file ppat.1006934.s001.tif]
